# From a Dense Structure to Open Frameworks: The Structural Plethora of Alkali Metal Iron Fluorophosphates

**DOI:** 10.1021/acs.inorgchem.2c01205

**Published:** 2022-06-14

**Authors:** Stefanie Siebeneichler, Katharina V. Dorn, Volodymyr Smetana, Alexander Ovchinnikov, Anja-Verena Mudring

**Affiliations:** Department of Materials and Environmental Chemistry, Stockholm University, Svante Arrhenius Väg 16 C, 10691 Stockholm, Sweden

## Abstract

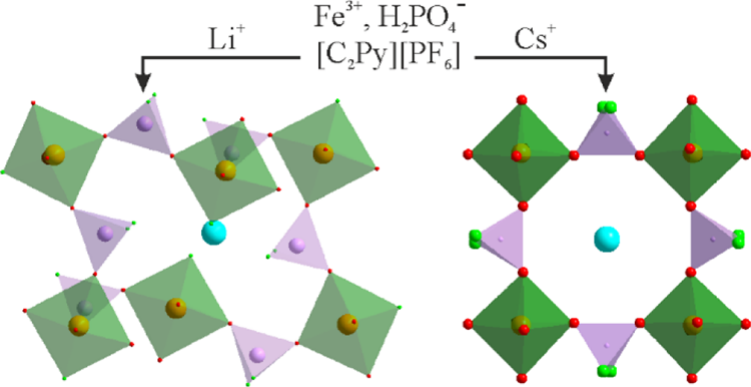

By employing the pyridinium hexafluorophosphate task-specific ionic liquids 1-butyl-4-methylpyridinium hexafluorophosphate ([C_4_mpyr][PF_6_]) and 1-ethylpyridinium hexafluorophosphate ([C_2_pyr][PF_6_]) as the reaction medium, mineralizer, structure-directing agent, and, in the case of the smaller pyridinium cation, even a structural component, it was possible to obtain five new alkali metal iron phosphates featuring interconnected FeX_6_ octahedra and PX_4_ (X = F, O, or OH) tetrahedra. NaFe(PO_3_F)_2_ (**1**) is a dense 3D structure, RbFe(PO_3_F)(PO_2_(OH)F)(PO_2_(OH)_2_) (**2**) features 1D strands, (C_2_pyr)LiFe(PO_3_F)_3_(PO_2_F_2_)F (**3**) has 2D layers, and LiFe(PO_3_F)(PO_2_F_2_)F (**4**) as well as Cs_0.75_Fe(PO_2.75_(OH)_0.25_F)(PO_2_F_2_)_2_ (**5**) are 3D open frameworks. While in **1–2** as well as in **4** and **5**, FeX_6_ octahedra and PX_4_ (X = F, O, or OH) tetrahedra alternate, **3** features octahedra dimers, Fe_2_X_11_ (X = F, O, or OH). The magnetic behavior of all compounds is governed by antiferromagnetic interactions. Interestingly, **3** exhibits a broad maximum in the temperature dependence of the magnetic susceptibility, characteristic of a low-dimensional magnetic system consistent with the presence of Fe–Fe dimers in its crystal structure.

## Abstract 2

Application of ionic liquids as the reaction medium, structure templates, and mineralizer led to a series of active-metal-iron phosphates with structural motifs of varied dimensionality and, ultimately, an open-framework structure.

## Introduction

Porous materials, such as zeolites,^1^ open-framework metal phosphates,^2^ metal–organic frameworks,^3^ covalent organic frameworks,^4^ and so on, are attracting considerable attention in both fundamental and applied research.^5,6^ Open-framework materials not only offer substantial structural diversity with spectacular architecture but also allow easy incorporation of additional components such as transition metal cations making it possible to further tune material properties such as magnetism,^7^ electrical transport,^8^ and electronic^9^ and optical^10^ properties. Furthermore, it is possible to combine different properties in one material and yield multifunctional materials with interesting property combinations.^11^ Such materials have also been extensively applied in industrial processes including absorption,^13^ ion exchange^14^, separation,^15^ heterogeneous catalysis^16^ and in oil refineries.^12^ In this context, particularly with transition metal phosphates,^17,18^ multifunctional materials can be realized, which combine porosity with magnetism, luminescence, or catalytic properties originating from a specific transition metal.^7,19,20^

While the metal phosphate frameworks belong to the most abundant group of the open-framework family and include a large variety of metal ions including transition and p block metals, their intensive explorations have begun only during the past decades.^21^ In this family, 3d transition metals Mn, Fe, Co, and Ni are the most interesting as they are earth-abundant and can offer exciting properties. For example, iron phosphates have been known as low-cost and environmentally friendly cathode materials for lithium-ion batteries.^22^ However, in contrast to other open-framework materials, laboratory synthesis of the open-framework metal phosphates remains challenging with the achieved cavity sizes remaining relatively small.^7,23−25^ Iron phosphates can form porous structures^26^ and a variety of such frameworks have been obtained in the laboratory by the hydrothermal approach.^27−32^ The obtained porous materials are particularly interesting due to their magnetism. For that reason, they have been investigated as magnetic sensors or magnetic separation media.^19,33,34^ It would be important to extend the exploration and particularly broaden the material base with new compounds. Those new materials may offer boundless possibilities with the ion choice and substitutions, their coordination environments, and the structure dimensionality.^11,35^

In this context, the successful application of the ionothermal route for the preparation of open-framework iron phosphates^36^ encouraged us to explore different combinations of inorganic cations and ionic liquids (ILs). Here, we report five new iron phosphates NaFe(PO_3_F)_2_ (**1a**), RbFe(PO_3_F)(PO_2_(OH)F)(PO_2_(OH)_2_) (**2**), (C_2_pyr)LiFe(PO_3_F)_3_(PO_2_F_2_)F (**3**), LiFe(PO_3_F)(PO_2_F_2_)F (**4**) and Cs_0.75_Fe(PO_2.75_(OH)_0.25_F)(PO_2_F_2_)_2_ (**5**). The obtained products vary in their architecture ranging between dense phosphates and layered materials with different degrees of segregation and truly open 3D frameworks. We observe that the realized Fe substructure has a pronounced effect on the magnetic exchange interactions and properties.

## Experimental Section

### Synthesis

To obtain compounds **1–5**, the respective starting materials were weighed and layered on top of each other inside of a 15 mL polytetrafluoroethylene vessel under an argon atmosphere in the following filling order: [C_4_mPy][PF_6_] (1-butyl-4-methylpyridinium hexafluorophosphate)—AH_2_PO_4_ (A = Li, Na, Rb, and Cs)–H_3_BO_3_–FeCl_3_ (**1a**, **1b**, **2**, **4**, and **5**) and AH_2_PO_4_ (A = Li and K)–[C_2_Py][PF_6_] (1-ethylpyridinium hexafluorophosphate)—H_3_BO_3_–FeCl_3_ (**3**) (Table S1, Scheme S1). It was noted that it was important to layer the starting material on top of each other in the respective order. If this was not done properly significant amounts of byproducts were obtained.

The reaction mixtures were heated to temperatures of 433 K (**1a**, **1b**, **2**, **4**, and **5**) and 453 K (**3**), with a heating rate of 1 K min^–1^, kept at this temperature for a week, and cooled to room temperature at a cooling rate of 0.03 K min^–1^ (**1a**, **1b**, **3–5**), by using ILs as both solvents (*T*_m_(Ils) = 318–379 K) and fluoride sources. RbFe(PO_3_F)(PO_2_(OH)F)(PO_2_(OH)_2_) (**2**) was quenched to room temperature after 7 d at 433 K. The transparent, colorless reaction products were purified by washing with deionized water and acetone. See Supporting Information for further details on the syntheses.

### Characterization

#### Powder X-ray Diffraction

Intensity data sets for powder X-ray diffraction of the title compounds were recorded by utilizing a Panalytical X’Pert PRO diffractometer with Cu Kα_1_ radiation (λ_1_ = 1.54059 Å) and a PIXcel detector (**1a**, **1b**, **2**, **3**, and **5**) and a Panalytical X’Pert PRO diffractometer with Cu Kα radiation (λ_1_ = 1.54059 Å, λ_2_ = 1.54443 Å) and a PW3015/20 X’Celerator detector (**4**) in Bragg–Brentano geometry at room temperature. Rietveld refinements (**1a** and **1b**) and Le Bail profile matching (**2**, **3**, and **5**) were employed using the Fullprof Suite^37^ (Figures S1–S3). As the synthesis of **4** yielded only very small sample sizes, the data quality did not allow performing either a Rietveld refinement or a Le Bail profile matching. The PXRD patterns of **1b**, **3,** and **4** contain no visible impurities. **1a** contains about 14(1) wt % of its monoclinic polymorph **1b**. In **2**, minor amounts of RbFe(PO_3_F)_2_ and RbFe_3_(PO_2_F_2_)_6_(PO_3_F)_2_(H_2_O)_*x*_ could be identified. Due to the strongly preferred orientations (needle- and plate-like crystallites), it was not possible to perform a satisfactory Rietveld refinement of the PXRD data. Nevertheless, from the relative peak intensities, the amount of each impurity was estimated to be less than 5 wt %. The PXRD pattern of **5** contains a number of low-intensity peaks that could not be attributed to the main phase or any other compound. We estimate this impurity to make up about 5 wt %.

#### Single-Crystal X-ray Diffraction

The crystal structural measurements of all compounds were carried out on a Bruker Venture diffractometer equipped with a Photon 100 CMOS detector and an IμS microfocus source using Mo Kα radiation (λ = 0.71073 Å) at room temperature. Intensity data of reflections were integrated using SAINT within the APEX3 software package.^38^ SADABS^39^ was used for the absorption corrections. The crystal structure solution was performed by intrinsic phasing using SHELXT.^40^ SHELXL^41^ was used for the subsequent difference Fourier analyses and least-squares refinement. All nonhydrogen atoms were refined anisotropically. The hydrogen positions have been calculated geometrically and refined using the riding model. Crystallographic data and details of structure refinements have been summarized in Table S4.

#### Magnetic Measurements

The magnetic properties were measured on a physical properties measurement system from Quantum design (USA). The vibrating sample magnetometer (VMS) option was used for temperature and field dependence in static (DC) fields at 0.1 T and up to 7 T, respectively. Polycrystalline samples were loaded into polypropylene capsules, which were mounted in a brass sample holder.

## Results and Discussion

### Structural Characterization

Crystalline samples of all compounds could be yielded via an ionothermal synthesis approach in which ILs were used as a solvent, structure-directing agent, and mineralizer supporting crystal growth.^7,25,27,42^ Single crystal X-ray diffraction (SCXRD) analyses reveal that NaFe(PO_3_F)_2_ (**1a**) crystallizes orthorhombically (SG *Fdd*2, *a* = 9.728(1) Å, *b* = 42.246(5) Å, *c* = 8.506(1) Å, *V* = 3495.4(8) Å^3^, and *Z* = 24) and is a polymorphic modification of the previously reported monoclinic analogue (**1b**).^43^ RbFe(PO_3_F)(PO_2_(OH)F)(PO_2_(OH)_2_) (**2**) is monoclinic (SG *P*2_1_/*c*, *a* = 7.645(1) Å, *b* = 14.801(3) Å, *c* = 9.518(2) Å, β = 107.962(6)°, *V* = 1024.5(3) Å^3^, and *Z* = 4), (C_2_pyr)LiFe(PO_3_F)_3_(PO_2_F_2_)F (**3**)—monoclinic (SG *P*2_1_/*c*, *a* = 21.8782(9) Å, *b* = 9.6563(4) Å, *c* = 19.0223(6) Å, β = 100.368(1)°, *V* = 3953.1(3) Å^3^, and *Z* = 8), LiFe(PO_3_F)(PO_2_F_2_)F (**4**)—orthorhombic (SG *P*2_1_2_1_2_1_, *a* = 6.4286(2) Å, *b* = 7.6240(3) Å, *c* = 13.8321(6) Å, *V* = 677.93(4) Å^3^, and *Z* = 4), and Cs_0.75_Fe(PO_2.75_(OH)_0.25_F)(PO_2_F_2_)_2_ (**5**)—tetragonal (SG *I*4/*mcm*, *a* = 18.159(2) Å, *c* = 12.874(2) Å, *V* = 4245.3(9) Å^3^, and *Z* = 16).

**Figure 1:**
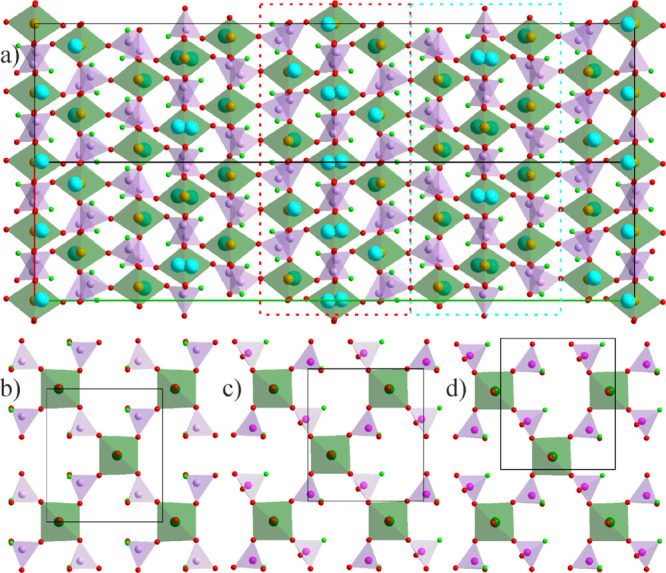
Projection of the crystal structure of **1a** on the *ab* plane (a). Three separate, consecutive phosphate layers in the crystal structure of **1a** along the *b* axis at *y* = 0.5, 0.577, and 0.673 (b, c, and d, respectively). Here, Fe atoms are orange, alkali metals—cyan, P—lavender, O—red, and F—light green. Fe octahedra are green and P tetrahedra are lavender. Crystallographic axes are color-coded: *a*—red, *b*—green, and *c*—blue. The dashed boxes outline the layers with isolated trimeric Na groups.

The crystal structure of NaFe(PO_3_F)_2_ (**1a**) is built up of distorted FeO_6_ octahedra and PO_3_F tetrahedra. The coordination environments of the two symmetry-independent Fe positions deviate slightly from octahedral symmetry with the Fe–O distances varying from 1.947(4) to 2.068(5) Å and 1.969(5) to 2.042(5) Å. The P–O distances across three symmetry-independent distorted PO_3_F tetrahedra range from 1.486(5) to 1.513(5) Å while the P–F distances from 1.565(5) to 1.574(5) Å. All interatomic distances agree well with that of previously reported fluorophosphates.^43−55^ The two symmetry-independent Na cations feature a 4 + 3 coordination. The short contacts to three O and one F position (*d*_Na–O_ = 2.27(1)–2.38(1) Å; *d*_Na–F_ = 2.26(1)–2.36(1) Å) create either distorted square planar or heavily squashed tetrahedral coordination environment for Na1 and Na2, respectively. The framework structure of **1a** can be visualized by layers of alternating O-vertex-sharing FeO_6_ octahedra and PO_3_F tetrahedra (Figure 1). The F atoms of the PO_3_F tetrahedra are not participating in the network, but coordinate with Na. The layers are linked via O-vertices with Fe–O–P connectivity in the third dimension (along the *b* axis). The tetrahedral arrangement of the Fe centers resembles a distorted diamond-type packing with honeycomb motifs (Figure S4). Similar transition metal networks were also observed in other phosphates such as Ba_2_*M*(PO_4_)_2_ (*M* = Mn and Ni).^56,57^ There are six inequivalent layers, which differ in the orientation of the PO_3_F groups and the positions of the octahedral and the tetrahedral units in the *ac* plane (Figure 1b–d). Their relative arrangement leads to the formation of prolonged cavities that extend over three layers accommodating the Na^+^ cations. The trimeric cationic groups in these cavities are parallel in the *ac* plane but have alternating orientations along the *b* axis (Figure 1a, red and blue dashed boxes). A minor positional disorder (split) of Na has been observed in the center of the cavity due to the local coordination environment and preference of Na for the lower coordination number (Figure S5).

**1a** exhibits structural similarities with its previously reported polymorph **1b**,^43^ including the arrangement of the Fe and P centers and isolated cationic (though dimeric) groups. In general, **1b** is a lower symmetric ordered variant of **1a**. The similarities between their local coordination environments, such as comparable Fe–P–Fe angles (Table S3), can be traced back to their three-dimensional metal-phosphate framework. However, the interlayer distances are as short as the intralayer spacings (*d*_intra_ = 4.743–5.158 Å) due to the three-dimensional topology. Both compounds even have comparable densities—3.133 (**1a**) versus 3.082 g·cm^–3^ (**1b**). The two polymorphs can be interpreted as stacking variants as analogous layers are present in the crystal structures of both polymorphs. The unit cell of **1b** can be transformed into that of **1a** by applying the transformation matrix (−10–1, 0–30, 10–1). However, no direct group–subgroup relationships could be observed. At first sight, the observation of polymorphism for **1** is puzzling as both of them form at the same reaction temperature, with the same IL and the same IL/Fe/P ratio. However, it was noted that increasing the H_3_BO_3_ ratio introduces a shift from **1b** being a single-phase product to becoming a minority phase and, at high concentrations, leading to the formation of **1a**. As the amount of H_3_BO_3_ regulates the fluoride concentration, this appears to be the structure-determining factor.

Like **1a**, the crystal structure of RbFe(PO_3_F)(PO_2_(OH)F)(PO_2_(OH)_2_) (**2**) features FeO_6_ octahedra. The FeO_6_ octahedra in the crystal structure of **2** are more regular compared to **1a** (*d*_Fe–O_ = 1.962(2)–2.024(3) Å). Another, more obvious, difference is the larger variety of phosphate and fluorophosphate building units in **2**. Due to the presence of three phosphate/fluorophosphate groups—PO_3_F, PO_2_(OH)F, and PO_2_(OH)_2_ and their involvement in the interchain hydrogen bonding, their shapes also differ. While in two latter −OH and −F groups are terminal, PO_3_F also contains one terminal O^2–^ participating in hydrogen bonding with two H-donor groups. The bridging P–O distances are in the range 1.481–1.507(3) Å, P–OH contacts—1.535–1.554(3) Å, and P–F—1.552–1.562(3) Å. The terminal P=O distance is 1.508(2) Å being at the upper edge for the bridging P–O contacts that can be attributed to the active involvement in multiple strong H bonds. The Rb cations are 10-coordinated while the coordination environment is highly irregular with big open faces due to stacking with neighboring Rb polyhedra. Both Rb–O and Rb–F contacts start from 2.96(2) Å, while the longest Rb–O contacts reach 3.387(3) Å.

**Figure 2:**
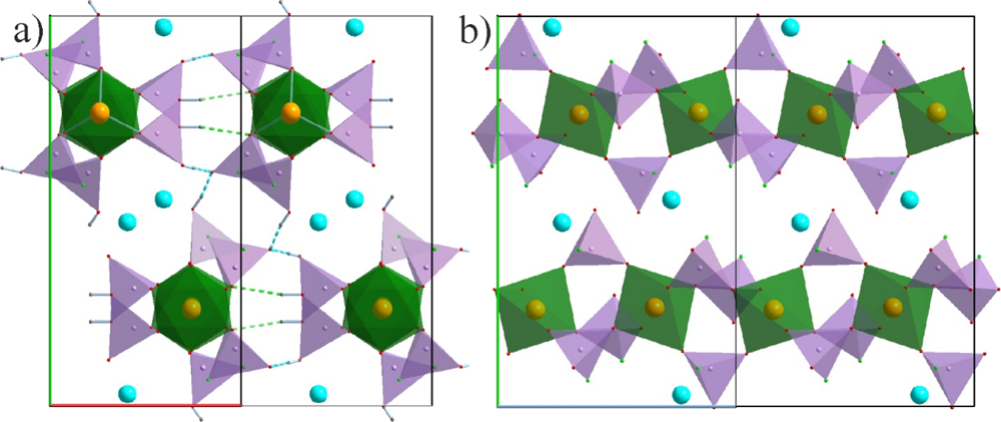
Projection of the crystal structure of **2** on the *ab* plane (a) and the *bc* plane (b). Hydrogen bonds are indicated with the dashed lines. Hydrogen positions in the right image have been omitted for clarity.

Taking into account only the connectivities between the octahedral FeO_6_ and tetrahedral PO_4–*x*_F_*x*_ units, the crystal packing appears to be 1-dimensional with quite isolated anionic chains and the cations filling the space in between. However, a closer inspection reveals a dense network of short hydrogen bonds resulting in a hydrogen-bonded 3D open framework with the Rb cations in the channels (Figure 2). FeO_6_ octahedra share their vertices with the phosphate groups (PO_3_F^2–^, HPO_3_F^–^, and H_2_PO_4_^–^), while the latter serve as bridges for the former. There are three phosphate bridges between the neighboring FeO_6_ octahedra leading to a chain extending down the *c* axis (Figure 2b). The connectivities between the chains are established solely via hydrogen bonding. The shortest OH···O bonds occur between the phosphate groups (*d*_OH···O_ = 1.61–1.69(3) Å) being complemented by weaker OH···O bonding involving, also, the FeO_6_ octahedra (*d*_OH···O_ = 2.12(3) Å). The terminal F positions do not participate in any hydrogen bonding. The Rb cations in the channels are well ordered and regularly distributed (*d*_Rb–Rb_ = 4.987(1) and 5.071(1) Å) forming zigzag chains. In general, connectivities in **2** resemble the picture observed in the family of Zr phosphates, for example, Zr[(NH_4_)_2_PO_4_]_2_F_2_·H_2_O and Zr(NH_4_PO_4_)[(NH_4_)_2_PO_4_]F·0.5H_2_O.^58^

The monoclinic Li representative (C_2_pyr)LiFe_2_(PO_3_F)_3_(PO_2_F_2_)F (**3**) is featuring FeO_5_F octahedra, fluorophosphate PO_3_F, PO_2_F_2_ tetrahedra, and LiO_4_ tetrahedra. The FeO_5_F octahedra are slightly distorted but there is no significant difference between the Fe–O and Fe–F contacts (1.929–2.028(7) and 1.957–1.964(4) Å). The longest Fe–O contacts [>2.02(1) Å] have been observed for the O position in the direct vicinity of Li. In contrast, the bridging F position has no active metals as possible electron donors leading to the shortening of the bond lengths being comparable to similar bonds in other iron fluorophosphates.^27^ The LiO_4_ tetrahedra are quite irregular with a relatively large spread in interatomic distances, (*d*_Li–O_ = 1.93–2.16(2) Å). P–O and P–F distances in the corresponding fluorophosphates tetrahedra are in the ranges 1.467–1.525(5) and 1.523–1.580(6) Å, respectively. It is rather usual that P–F contacts are longer, but it is worth noting that all of them are directed toward the organic layers participating in weak electrostatic CH···F interactions (*d*_F–H_ = 2.35–2.68(3) Å), slightly beyond the commonly accepted range for the hydrogen bonds.^59^ Such weak connectivity can particularly explain the low crystallinity of the examined single crystals.

**Figure 3:**
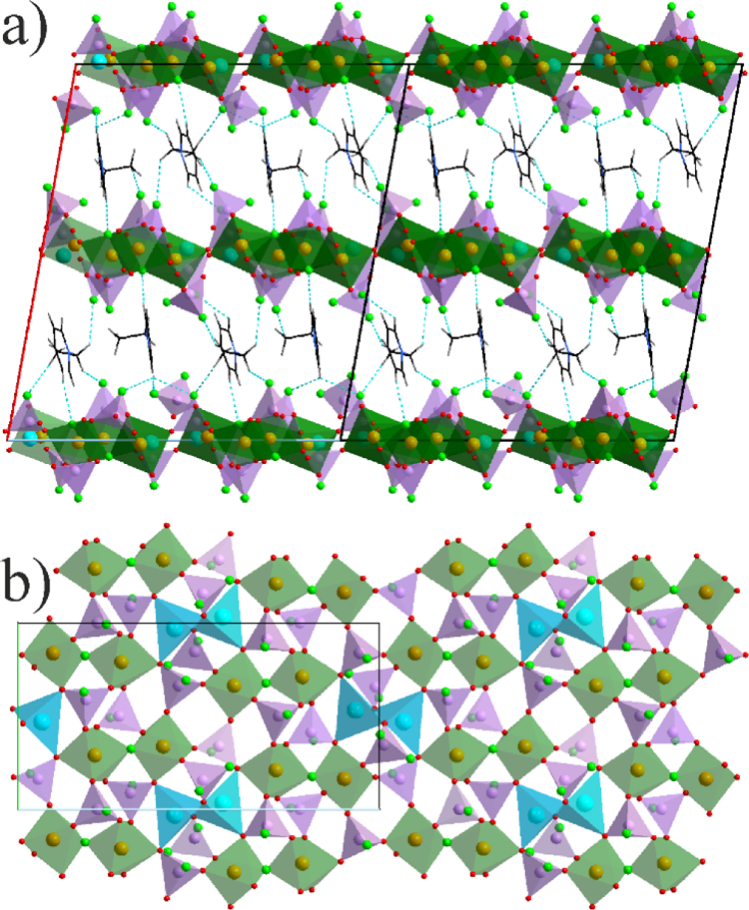
Projection of the crystal structure of **3** on the *ac*- (a) and the inorganic layer on the *bc* plane (b).

Compared to the other compounds reported here, **3** stands out with a unique structure and composition: The common FeO_6_ octahedra are substituted by dimeric FeO_5_–F–FeO_5_ units—double octahedra sharing a common F vertex, while the fluorophosphates groups are represented by PO_3_F and PO_2_F_2_. There are two types of charge balancing cations with distinct roles in the structure—Li fills the voids within the phosphate layers, while C_2_pyr cations serve as bridges for the latter, forming separate organic layers. Both layers stack along the *a*-axis (Figure 3). A similar but more complex layer separation has been observed in the crystal structure of another phosphate [C_6_N_4_H_21_][Fe_2_F_2_(HPO_4_)_3_][H_2_PO_4_]·2H_2_O with an organic countercation.^60^ The C_2_pyr cations are oriented practically orthogonal to the phosphate layers. Li cations in the cavities are tetrahedrally coordinated and also form pairs in form of edge-sharing LiO_4_ tetrahedra (Figure 3b, blue). Their role is, in part, similar to the transition metal ions, and they can also be considered as the layer forming blocks. None of the blocks form independent infinite motifs but a combination of them. For example, chains of the dimeric Fe and Li units can be outlined (Figure 3b, blue and green polyhedra). The phosphate groups serve as bridges binding everything into a layer. PO_3_F groups bind three FeO_5_F octahedra (two of them from one pair), while PO_2_F_2_ binds FeO_5_F with the LiO_4_ tetrahedral pairs.

The crystal structure of the second Li representative LiFe(PO_3_F)(PO_2_F_2_)F (**4**) as well as the Cs compound—Cs_0.75_Fe(PO_2.75_(OH)_0.25_F)(PO_2_F_2_)_2_ (**5**) contain identical fluorophosphate building units—PO_3_F and PO_2_F_2_. The octahedral units are slightly different—FeO_6_ in **5** and FeO_5_F in **3** due to the additional bridging F position in the latter leading to dimeric FeO_5_–F–FeO_5_ units with a common vertex similar to **3**.

FeO_5_F octahedra in **4** are slightly distorted and, similar to **3,** show no clear differentiation between Fe–F and Fe–O connectivity (1.958(2) and 1.935–2.012(2) Å, respectively). P–O and P–F distances show certain segregation typical for all other fluorophosphates—1.491–1.518(2) and 1.555–1.563(3) Å, respectively. Typically, all F positions are terminal and are responsible for the cation’s connectivity. The Li cations are tetrahedrally coordinated—Li@OF_3_. It is revealed that the Li–F distances to the F positions bridging multiple cations (incl. Fe^3+^) are shorter (1.82–1.86(1) Å) than to O and F from the fluorophosphate groups (2.00–2.16(1) Å). In this light, Li^+^ in **4** stays closer to C_2_pyr^+^ in **3** showing limited connectivity with the P–O groups.

FeO_6_ octahedra in the crystal structure of **5** are almost ideal (*d*_Fe–O_ = 1.976–1.992(2) Å), while PO_2_F_2_ and PO_3_F tetrahedra show some diversity due to positional disorders, making precise analysis a little complicated. However, O and F positions could clearly be distinguished by the distance criterion. For example, P–O and P–F distances in the fully ordered PO_2_F_2_ group are 1.490(2) and 1.554(4) Å. A very similar tendency is also observed as an average picture for the disordered groups. The most interesting situation was observed for the PO_3_F group where one O position is terminal. The major disorder component shows the P–O distance of 1.45(1) Å that can be interpreted as a P=O bond,^61^ while there is a possibility for much longer contacts—1.65(3) Å. Taking into account the total charge balance due to partially occupied Cs positions, we concluded that a smaller part of those disordered O positions must be protonated compensating for the missing positive charge of 0.25/f.u., providing an explanation for the longer distances. Partial occupation of Cs as refined from SCXRD data also perfectly match with the EDX results—suggesting a Cs/Fe ratio of 0.73(2). All Cs positions are 16-coordinated and the coordination polyhedron can be described as 8-fold equatorially capped tetragonal prism—Cs@O_8_F_8_.

**Figure 4:**
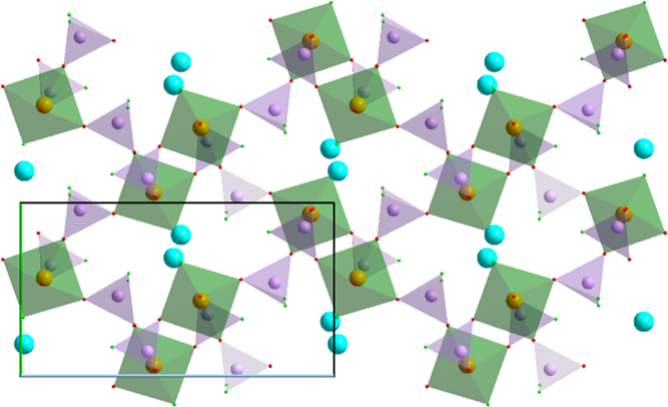
Projection of the crystals structure of **4** on the *bc* plane.

**Figure 5:**
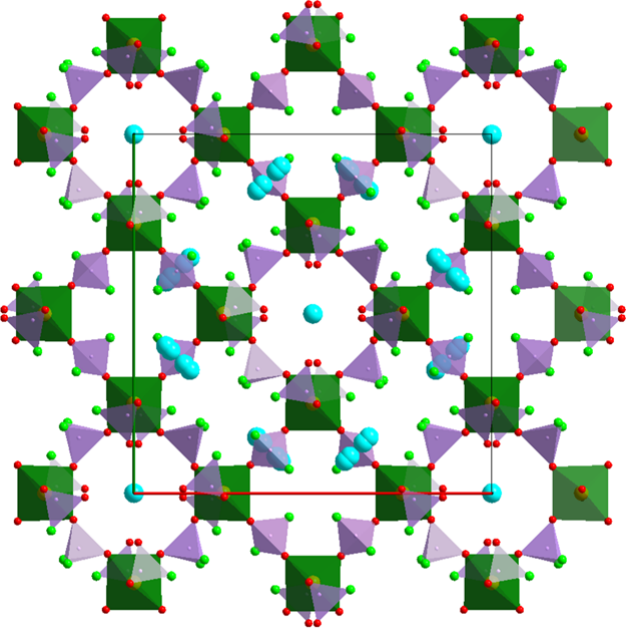
Projection of the crystal structure of **5** on the *ab* plane. The minor disorder components of the phosphate groups have been omitted for the sake of clarity.

Both **4** and **5** represent 3D open frameworks. Despite such a significant difference in the cation’s size, the structure building principles are astonishingly similar, although the degree of openness is different. The latter is expressed in the directionality of the tunnels extending solely along the *a*-axis in **4** (Figure 4) and in the three orthogonal directions in **5**, namely one along the *c*-axis and two parallel to the face diagonals of the *ab* plane (Figure 5). Both structures contain channels (with 12 and 8-ring openings in **4** and **5**, respectively); however, the distribution of cations varies. It is interesting that in **5**, despite the practically isotropic structure of the tunnels in all directions, no cubic symmetry is detectable .

Despite being small and practically invisible on the projection, the tunnels in **4** follow a zigzag shape due to the relative positions of the Fe octahedra and are quite large for comparatively small Li^+^. Similarly to **3**, FeO_5_F, PO_3_F, and PO_2_F_2_ are the main building blocks of the structure. Coordination of Li^+^ cations is also tetrahedral in accordance with its size, though a strong F preference is observed (LiOF_3_) as all terminal F^–^ from all of the building blocks are directed toward Li^+^. It is also worth noting that the terminal F of the FeO_5_F octahedra serves as a bridge between Fe and two Li positions.

Due to its large size, Cs^+^ acts more actively as the framework-directing agent. This is easily detectable in the incompatibility of the tunnel and the cation dimensions leading to extensive positional disorders. Cs positions occupy the largest possible cavities in the tunnel located between four FeO_6_ octahedra and four PO_2_F_2_/PO_3_F groups being restricted from the above and below by four other fluorophosphate groups, though leaving enough freedom along the corresponding axes. This freedom consequently leads also to the disorder of the corresponding fluorophosphate groups. The distribution and orientation of the disordered fluorophosphate groups, particularly O and F positions give a hint about the missing higher symmetry. In all channels, Cs^+^ cations are located around bigger O_8_ openings formed by edges of four neighboring FeO_6_ octahedra; however, separators or smaller openings differ. In the channels extending parallel to the *ab* plane, these are solely terminal F positions of the PO_2_F_2_ tetrahedra, while in those going along the *c* axis—terminal partially protonated O. Consequently, the Cs disorder within each type of channel is also different both together leading to the tetragonal distortion of the unit cell.

The dimensionality of the structural motifs in **1–5** varies from chains to layers and frameworks and can be followed analytically with the ∠Fe–P–Fe angle changes, though with a reservation for special structural features. In the layered AFe(PO_3_F)_2_ (A = K, NH_4_, Rb, and Cs) compounds, the radial arrangement of the [PO_3_F] tetrahedra around the [FeO_6_] octahedra is characterized by ∠Fe–P–Fe ranging from 97.48 to 110.21°.^62^ In **1a** and **1b**, the frameworks are built up by two sets of angles: the first ones are comparable to the ones observed in AFe(PO_3_F)_2_ (∠Fe–P–Fe = 93.35–101.56(2)°); the second ones, however, are significantly larger with ∠Fe–P–Fe = 153.48–155.22(3)°. Similarly, two groups of angles (93.38–93.46(2) and 130.96(3)°) have been observed also in **4** with an open framework, while all Fe–P–Fe angles in **5** are large (153.49–156.42(3)°). The latter framework is more isotropic with a higher degree of openness. In contrast, all angles in the 1D compound **2** are small (91.64–92.49(2)°). The layered structure of **3** cannot be compared directly due to Fe dimers bringing an additional Fe–O–Fe connectivity; however, all Fe–P–Fe angles not involved in this pair formation are in the standard range for the lower-dimensional formations (96.40–103.46(5)°). However, the enhanced connectivity between the dimers leads to extra smaller (68.62(4)°, within one pair) and larger (126.16(6)°, between two pairs) angles.

### Magnetic Properties

Temperature- and field-dependent magnetization studies were performed to examine the magnetic exchange in the reported iron phosphates. Phase-pure **1a** could not be obtained, samples always contained both polymorphs. However, phase-pure **1b** could be obtained which allowed for studying its magnetism. Unfortunately, the sample yield of **4** was too low to allow for magnetic characterization.

**Figure 6:**
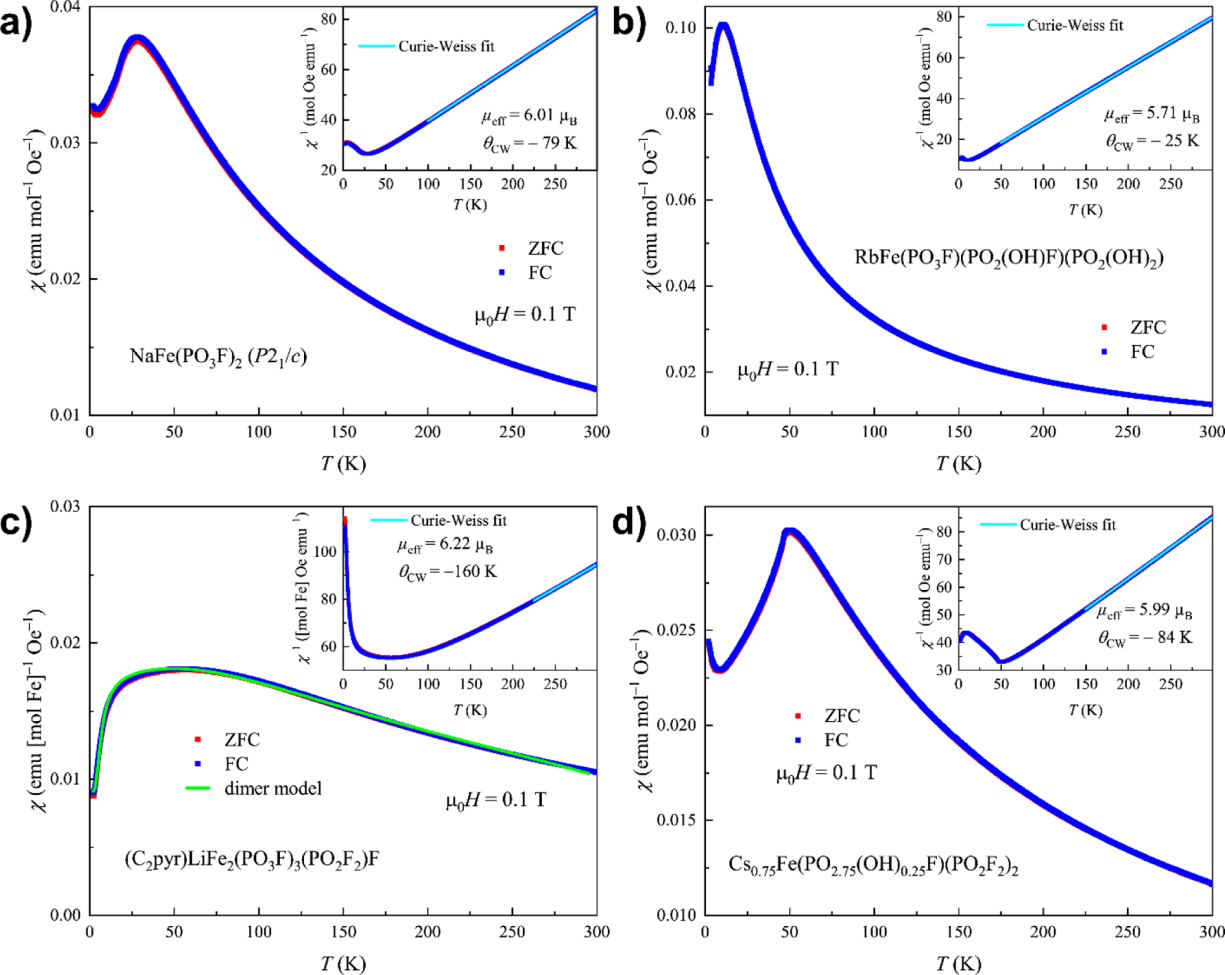
Temperature dependence of magnetic susceptibility for **1b** (a), **2** (b), **3** (c), and **5** (d). Insets show Curie–Weiss fits of the inverse susceptibility. Red and blue curves denote ZFC and FC data, respectively. The green curve is the best fit with eq 1.

All examined materials demonstrate localized paramagnetism at high temperatures, as can be seen from the temperature evolution of the magnetic susceptibilities (Figure 6). Curie–Weiss fits of the inverse susceptibilities χ^–1^(*T*) yield effective magnetic moments of 5.71–6.22 μ_B_, in fair agreement with the theoretical value of 5.92 μ_B_ for the high-spin Fe^3+^ (*S* = 5/2). The magnetic exchange appears to be dominated by antiferromagnetic (AFM) interactions, as indicated by the negative values of the Weiss constants, Θ_CW_. Fisher’s heat capacity, d(χ*T*)/d*T*, for all materials is given in Figure S6.

In **1b** (Figure 6a), an AFM ordering is observed below *T*_N_ = 18.5 K. The zero-field-cooled (ZFC) and field-cooled (FC) magnetic susceptibility curves, χ(*T*), do not exhibit any bifurcation, suggesting the absence of spin canting in the magnetically ordered state. The Weiss constant, Θ_CW_ = −79 K, extracted from the Curie–Weiss fit, is greater in magnitude than the observed ordering temperature, indicating some magnetic frustration, which can be quantified by the frustration parameter *f* = |Θ_CW_|/*T*_N_ ≈ 4 (for a nonfrustrated system, *f* ≈ 1). We note, however, that the frustration parameter cannot distinguish between different mechanisms of magnetic order suppression, such as geometric frustration or reduced magnetic dimensionality. In addition, the magnitude of *f* should be taken as a semi-quantitative measure.

An AFM transition is observed for RbFe(PO_3_F)(PO_2_(OH)F)(PO_2_(OH)_2_) (**2**), which enters an ordered state below *T*_N_ = 7.0 K (Figures 6b and S6). In this case, given the absolute value of the Weiss constant, Θ_CW_ = −25 K, the frustration parameter *f* amounts to about 3.5. Just like in the case of NaFe(PO_3_F)_2_, the magnetic moments in the ordered state appear to be perfectly compensated. It has to be noted that **2** contained minor amounts of two potentially magnetic impurities; however, not at least due to their low proportion, we did not observe any additional transitions. For instance, the major impurity RbFe(PO_3_F)_2_ must undergo a broad, AFM transition at 8.4 K.^62^

In contrast to **1b** and **2**, exhibiting well-defined transitions to the magnetically ordered state, (C_2_pyr)LiFe_2_(PO_3_F)_3_(PO_2_F_2_)F (**3**) demonstrates a broad maximum in the temperature dependence of the magnetic susceptibility, characteristic of a low-dimensional magnetic system (Figure 6c). Because the crystal structure of **3** features Fe dimers, which could be responsible for the observed magnetic behavior, we attempted to fit the χ(*T*) data using the theoretical expression for an *S* = 5/2 dimer system (first term in eq 1).^63^ However, this did not result in a satisfactory fit. Analysis of the χ(*T*) behavior under different applied fields uncovered weak field dependence of χ(*T*) even in the paramagnetic region (Figure S7a), pointing toward the presence of a small amount of ferromagnetic impurity in the sample. To take this contribution into account, we added an exponential term, representing the critical exponent behavior of a ferromagnet, to the fitting function (second term in eq 1). The best fit, shown in Figure 6c, reproduces the temperature evolution of the magnetic susceptibility fairly well. We note that the dimer expression used for the fitting assumes that (a) the magnetic dimers in the structure are equivalent and (b) the atoms building a dimer are related by symmetry. In fact, none of these assumptions are satisfied for **3**, which may be the reason for the observed small deviations between the fitted curve and the experimental data. The dimer and ferromagnetic contributions are plotted separately in Figure S7b,c.

The magnetic exchange parameter *J* (which should be taken as an average value for the nonequivalent types of dimers in the structure) measures −20.2 K (i.e., −14.0 cm^–1^), indicating moderately strong AFM exchange within the dimers.^63^
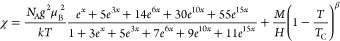
1where *x* = *J*/*kT*, *N*_A_ and *k* are the Avogadro and Boltzmann constant, respectively, *J* is the exchange coupling within the dimers, *g* is the Landé factor, μ_B_ is Bohr magneton, *H* is the applied field, *M* is the saturated magnetic moment of the ferromagnetic impurity, *T*_C_ is its Curie temperature, and β is the ferromagnetic critical exponent.

Analysis of the high-temperature portion of the χ(*T*) curve indicates strong AFM interactions, as can be judged from the large negative Weiss constant, Θ_CW_ = −160 K. Although this value should be taken with care, because the data are somewhat affected by the ferromagnetic contribution, the strong short-range AFM exchange is expected from the presence of the magnetic dimers.

Finally, the temperature dependence of the magnetic susceptibility for Cs_0.75_Fe(PO_2.75_(OH)_0.25_F)(PO_2_F_2_)_2_ (**5**) reveals a sharp maximum at *T*_N_ = 46 K, consistent with AFM ordering below this temperature (Figure 6d). With the fitted Weiss constant Θ_CW_ of −84 K, the frustration parameter *f* = |Θ_CW_|/*T*_N_ reaches a value of only about 1.8, indicating no sizeable magnetic frustration. Interestingly, despite the considerable crystallographic disorder and the shortest Fe–Fe distance exceeding 6.4 Å, the ordering temperature of **5** is significantly higher than that of other fluorophosphates studied here. A likely explanation for this behavior is the more isotropic pattern of Fe–Fe distances in all three dimensions, as found in the crystal structure of **5**. Indeed, while the Fe substructures in **1b**, **2**, and **3** clearly display reduced dimensionalities, the arrangement of Fe atoms in the crystal structure of **5** represents an almost perfect simple cubic packing. Apparently, the disorder in the nonmagnetic part of the structure does not inhibit superexchange significantly. All this ultimately results in the observed high temperature of the magnetic transition.

## Conclusions

Five iron fluorophosphates have been synthesized with the ionothermal approach using the ILs [C_2_Py][PF_6_] or [C_4_mPy][PF_6_] as the solvent and mineralizer. Their crystal structures have been established using SCXRD. All compounds feature interconnected FeX_6_ octahedra and PX_4_ (X = F, O, and OH) tetrahedra as building units, but with different secondary structures ranging from chains to 3D networks. NaFe(PO_3_F)_2_ is a dense phosphate; RbFe(PO_3_F)(PO_2_(OH)F)(PO_2_(OH)_2_) exhibits 1D chains; (C_2_pyr)LiFe(PO_3_F)_3_(PO_2_F_2_)F consists of iron-phosphate layers separated by the cations, while LiFe(PO_3_F)(PO_2_F_2_)F and Cs_0.75_Fe(PO_2.75_(OH)_0.25_F)(PO_2_F_2_)_2_ exhibit true open frameworks with the cations filling the tunnels. The last two feature 8-membered ring channels extending along [100] in the former or [001], [110], and [1–10] in the latter. The interconnection of the octahedral units in all compounds goes through various P(O,F,OH)_4_ tetrahedral units, while (C_2_pyr)LiFe_2_(PO_3_F)_3_(PO_2_F_2_)F is the only exception where direct connectivity is established through μ_2_-F bridges leading to Fe dimers. The presence of OH groups in RbFe(PO_3_F)(PO_2_(OH)F)(PO_2_(OH)_2_) is crucially important from the structural point, converting these formally isolated iron-phosphate chains into hydrogen-bonded open frameworks. Exchange interactions of predominantly AFM type govern the magnetism in the studied fluorophosphates, with the varying topologies of the Fe substructure affecting the magnetic behavior. Most of these compounds undergo long-range AFM ordering at low temperatures, and the ordering temperature shows certain correlation with the Fe lattice dimensionality being the highest in the isotropic 3-dimensional Cs_0.75_Fe(PO_2.75_(OH)_0.25_F)(PO_2_F_2_)_2_. (C_2_pyr)LiFe_2_(PO_3_F)_3_(PO_2_F_2_)F exhibits pronounced low-dimensional magnetism, which makes it an interesting system for further studies.

## Supporting Information Available

Additional synthesis details, crystallographic information, and magnetic data (PDF)

## Accession Codes

CCDC 2141973–2141977 contain the supplementary crystallographic data for this paper. These data can be obtained free of charge via www.ccdc.cam.ac.uk/data_request/cif, or by emailing data_request@ccdc.cam.ac.uk, or by contacting The Cambridge Crystallographic Data Centre, 12 Union Road, Cambridge CB2 1EZ, UK; fax: +44 1223 336033.
