# Supplementary material for: From a Dense Structure to Open Frameworks: The Structural Plethora of Alkali Metal Iron Fluorophosphates
Source: Inorg Chem. 2022 Jun 14;61(25):9767–75. doi: 10.1021/acs.inorgchem.2c01205 (PMC9490823; doi:10.1021/acs.inorgchem.2c01205)
Supplement: Supplementary file 1 — ic2c01205_si_001.pdf [file ic2c01205_si_001.pdf]

From a dense structure to open frameworks: The structural plethora of alkali metal iron fluorophosphates

Stefanie Siebeneichler, Katharina V. Dorn, Volodymyr Smetana, Alexander Ovchinnikov, Anja-Verena Mudring*

Department of Materials and Environmental Chemistry, Stockholm University, Svante Arrhenius väg 16 C, 10691 Stockholm, Sweden.

Supporting Information

Table S1. Synthesis details for compounds **1** – **5**

	***m*, g**	***n*, mmol**	***m*, g**	***n*, mmol**	***m*, g**	***n*, mmol**	***m*, g**	***n*, mmol**	**molar ratio**	**T_{max}, °C**
**1a**	$[C_4mPy][PF_6]$		$FeCl_3$		NaH_2PO_4		H_3BO_3			
	0.520	1.76	0.243	1.50	0.240	2.00	0.093	3.0	3.6:3.0:4.0:3.0	160
**1b**	$[C_4mPy][PF_6]$		$FeCl_3$		NaH_2PO_4		H_3BO_3			
	0.520	1.76	0.243	1.50	0.240	2.00	0.031	0.50	3.6:3.0:4.0:1.0	160
**2**	$[C_4mPy][PF_6]$		$FeCl_3$		RbH_2PO_4		H_3BO_3			
	0.520	1.76	0.243	1.50	0.365	4.00	0.108	3.5	3.6:3.0:4.0:3.5	160
**3**	$[C_2Py][PF_6]$		$FeCl_3$		LiH_2PO_4		H_3BO_3			
					0.104	1.00				
	0.523	2.1	0.251	1.55	KH_2PO_4		0.094	1.52	4.2:3.0:2.0:2.0:3.0	180
					0.136	1.0				
**4**	$[C_4mPy][PF_6]$		$FeCl_3$		LiH_2PO_4		H_3BO_3			
	0.520	1.76	0.243	1.50	0.208	2.00	0.093	1.50	3.6:3.0:4.0:3.0	160
**5**	$[C_4mPy][PF_6]$		$FeCl_3$		CsH_2PO_4		H_3BO_3			
	0.520	1.76	0.243	1.50	0.460	2.00	0.108	1.75	3.6:3.0:4.0:3.5	160

Table S2. Purity and supplier of the starting materials.

	Starting material	Sum formula	Purity	Supplier
**Ionic Liquid**	1-ethylpyridinium hexafluorophosphate	$[C_2Py][PF_6]$	99 %	IOLITEC
	1-butyl-4-methylpyridinium hexafluorophosphate	$[C_4mPy][PF_6]$		Tokyo Chemical Industry
**Metal source**	iron(III)chloride	$FeCl_3$	> 98 %	Merck
**Phosphate source**	lithium dihydrogen phosphate	LiH_2PO_4	99 %	Sigma-Aldrich
	potassium dihydrogen phosphate	KH_2PO_4	≥ 99 %	Scharlau
	orthophosphoric acid 85 %	H_3PO_4	85.6 %	VWR
	boric acid	H_3BO_3	≥ 99.5 %	Honeywell
**Solvents**	acetone	C_3H_6O		
	methanol	CH_3OH	≥ 99.8 %	
**Alkali carbonates**	rubidium carbonate	Rb_2CO_3	>99.975 %	Alfa Aesar
	caesium carbonate	Cs_2CO_3	99.99 %	Acros Organics

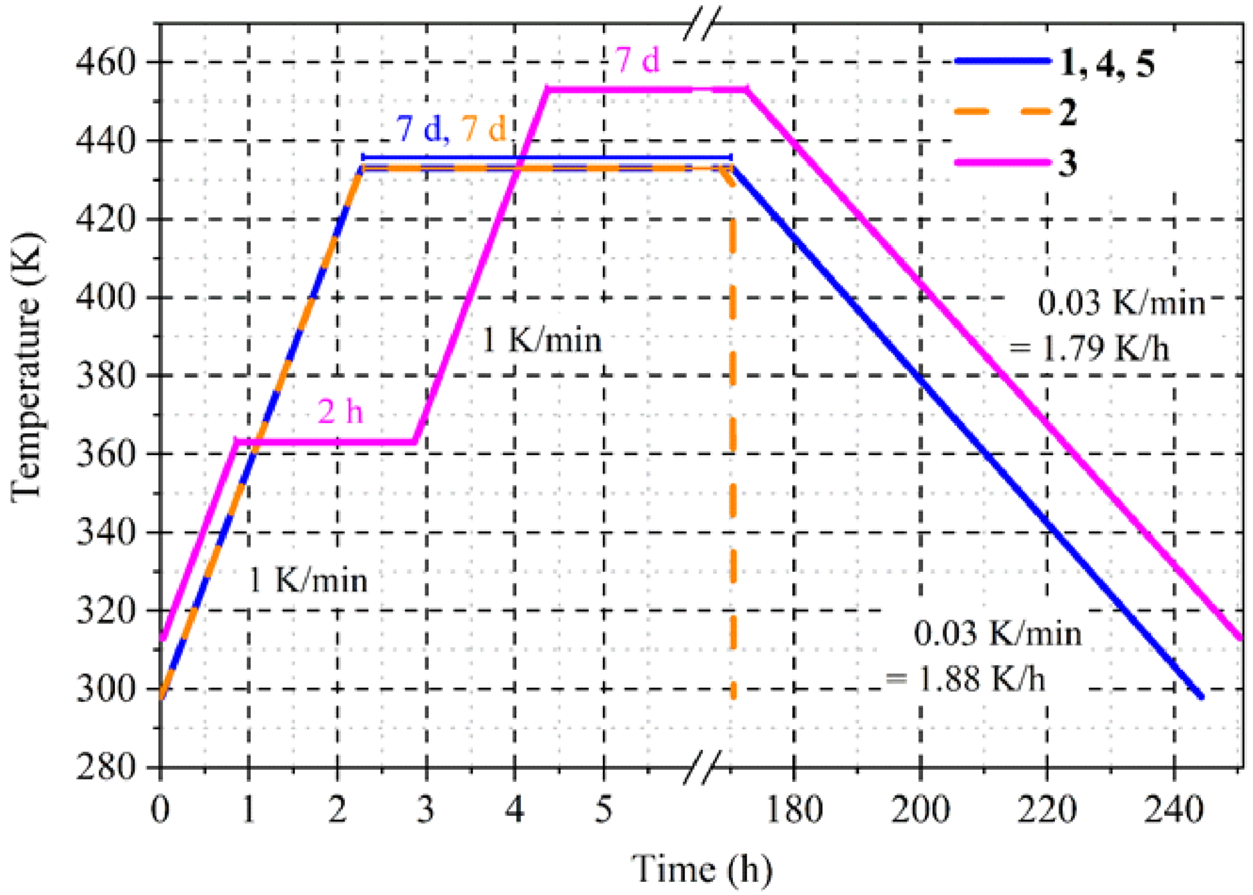

Scheme S1. Temperature program for the syntheses of the title compounds **1**, **2**, **4**, and **5** *(*T_{max} = 433 K), and **3** (T_{max} = 453 K). Except for **2**, which was quenched to room temperature, all other compounds were cooled slowly.

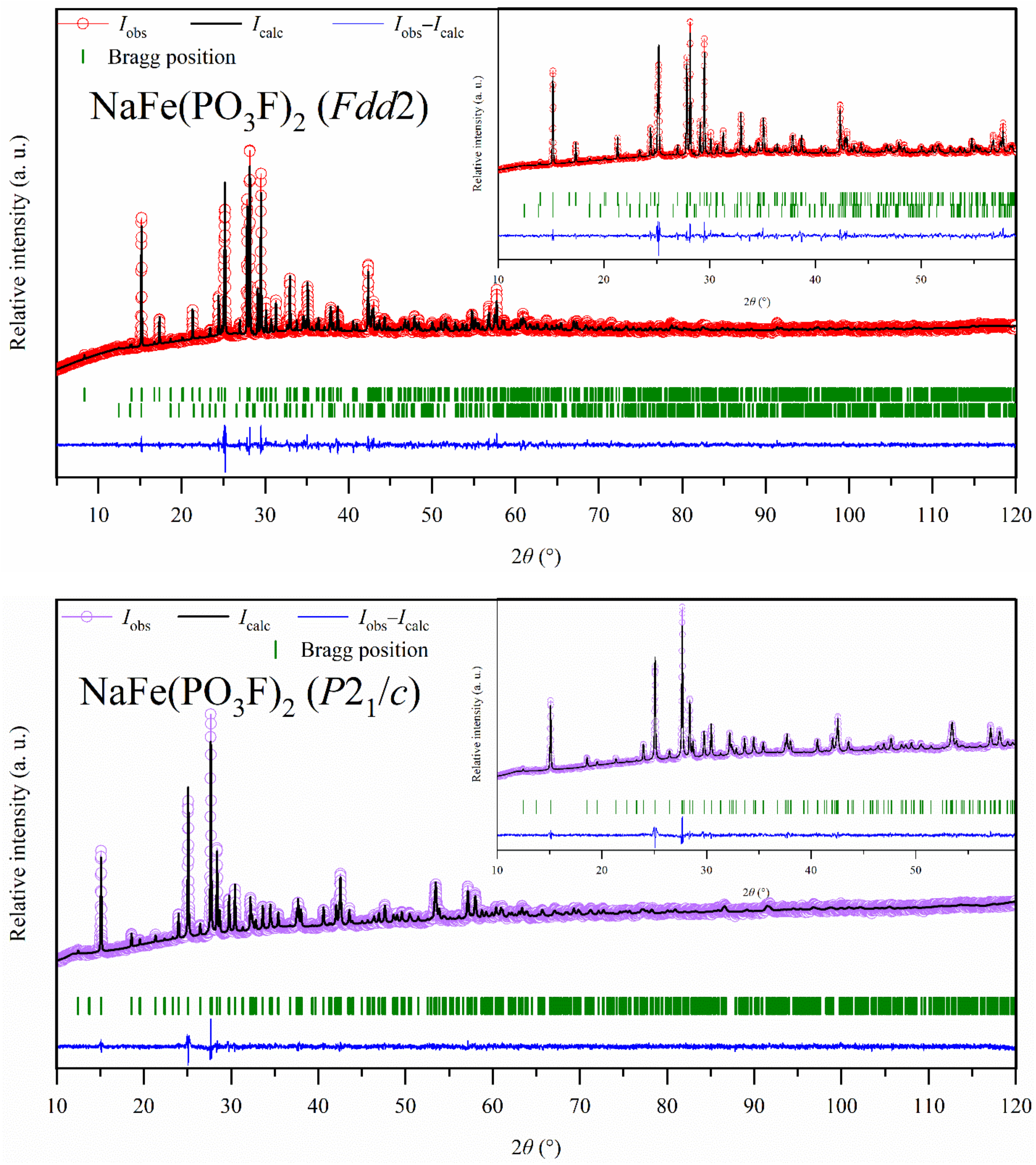

Figure S1. RIETVELD refinements of the PXRD patterns of the orthorhombic (*Fdd*2, **1a**, *top*) and monoclinic modifications ($P2_1/c$, **1b**, *bottom*) of $NaFe(PO_3F)_2$ in the 2θ-range between 5 – 120° and 10 – 120°, respectively. The inset shows a zoom-in of the region from 10 to 60°. Although **1b** could be synthesized single-phase, the samples containing **1a** also always contained significant fraction of **1b** (*bottom row Bragg markers*).

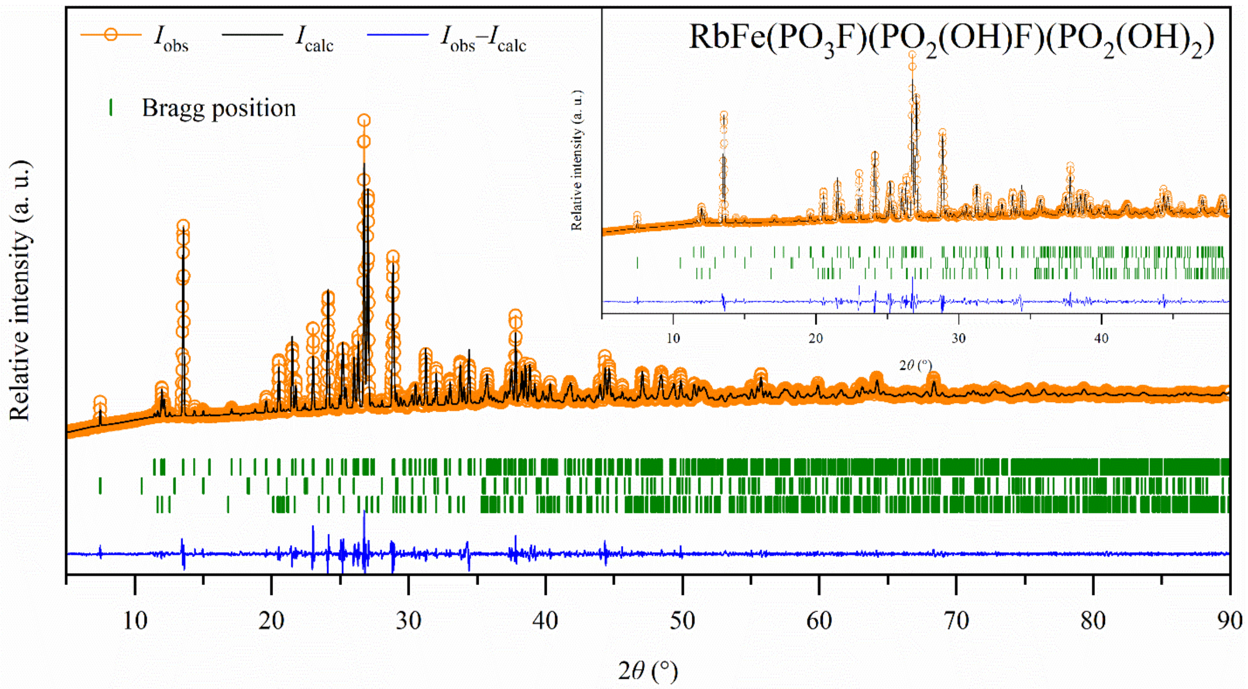

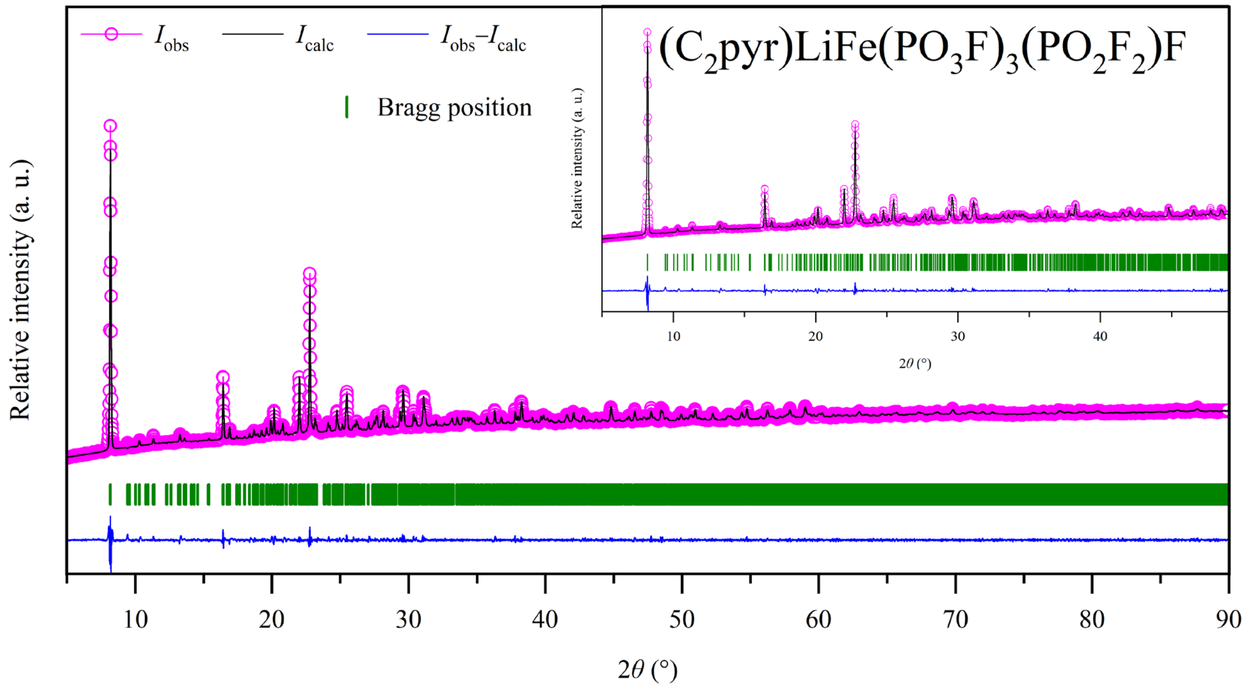

Figure S2. LE BAIL PXRD profile-fitting of RbFe(PO$_3$F)(PO$_2$(OH)F)(PO$_2$(OH)$_2$) (**2**, *top*) and (C$_2$pyr)LiFe(PO$_3$F)$_3$(PO$_2$F$_2$)F (**3**, *bottom*) in the 2θ-range between 5 – 120°. The inset shows a zoom-in of the region from 5 to 50°. **2** could not be synthesized entirely single-phase containing minor amounts of the impurities, i.e. RbFe(PO$_3$F)$_2$ (*bottom row Bragg markers*) and RbFe$_3$(PO$_2$F$_2$)$_6$(PO$_3$F)$_2$(H$_2$O)$_x$ (*middle row Bragg markers*).

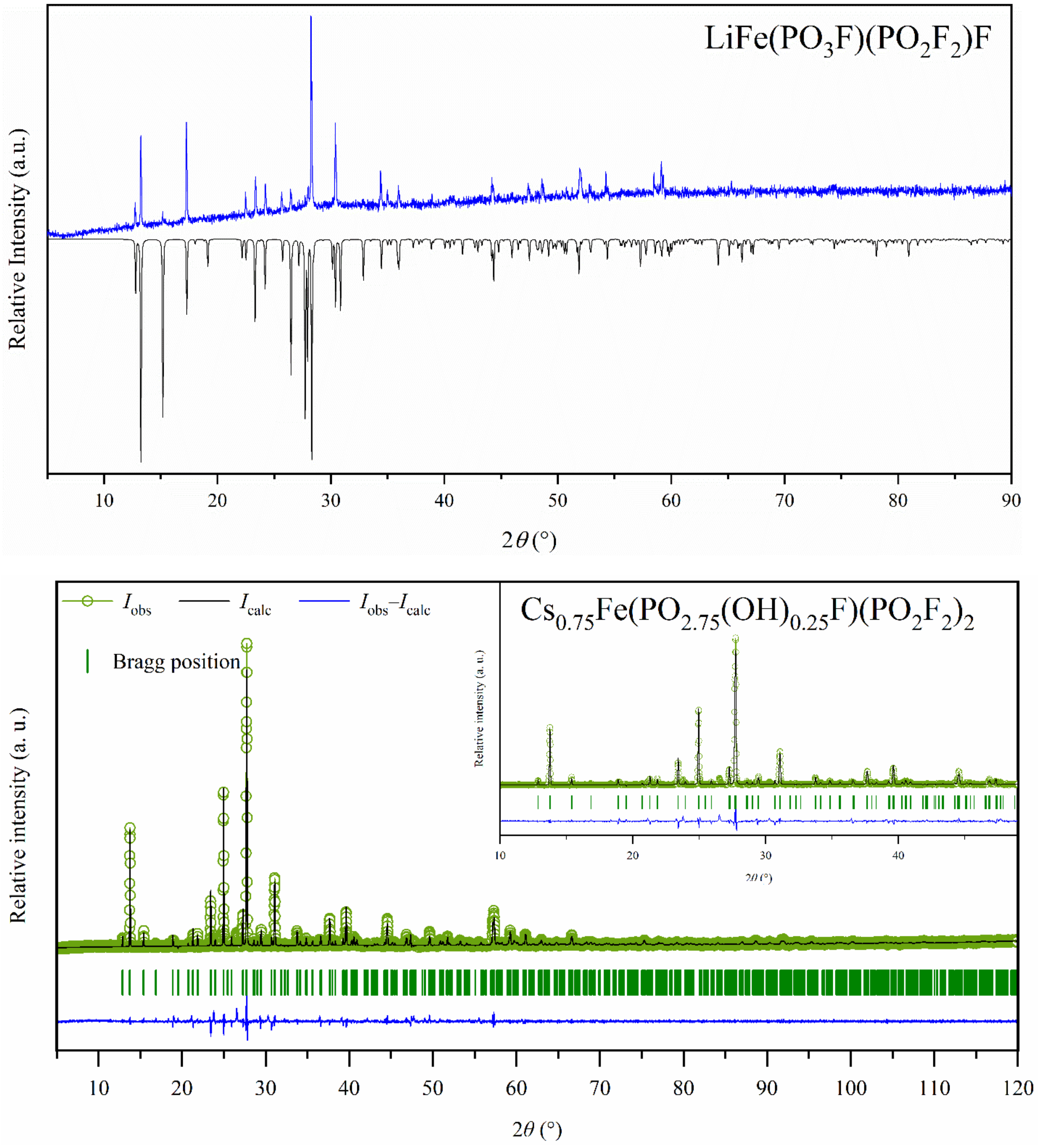

Figure S3. A comparison of the PXRD pattern of **4** with the theoretical pattern derived from single-crystal refinements in the 2θ-range between 5 – 90° (*top*). The difference in relative intensities is attributed to preferred orientation as the crystals have the shape of needles. LE BAIL PXRD profile-matching of $Cs_{0.75}Fe(PO_{2.75}(OH)_{0.25}F)(PO_2F_2)$ (**5**, *bottom*) in the 2θ-range between 5 – 120°. The inset shows a zoom-in of the region from 5 to 50°. **5** contains small amount of an unidentified impurity.

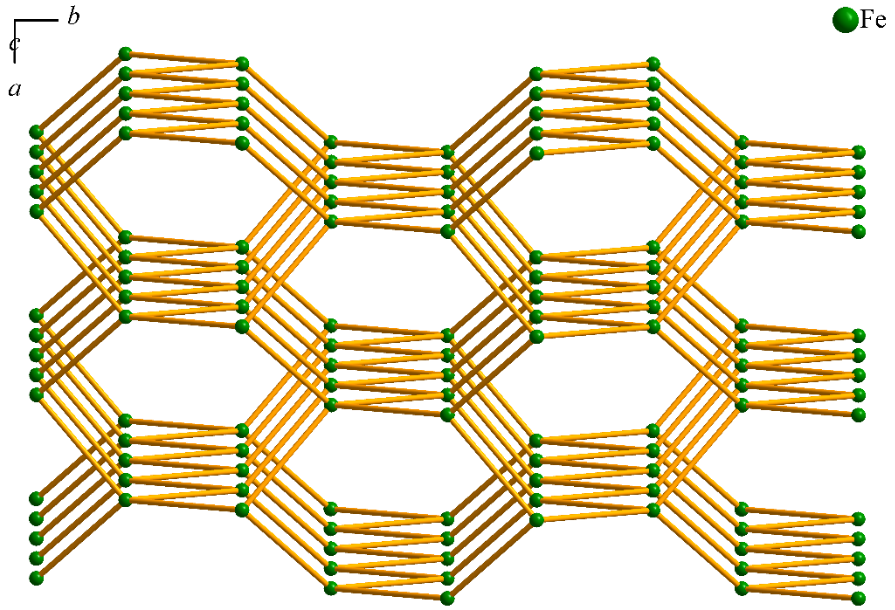

Figure S4. Fe network in the crystal structure of **1a**.

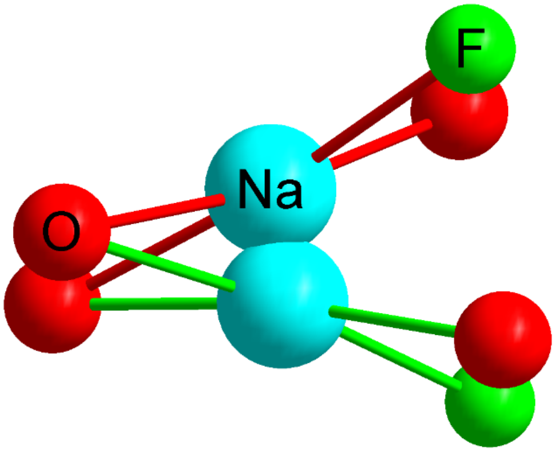

Figure S5. Local environment around the disordered Na position in **1a**.

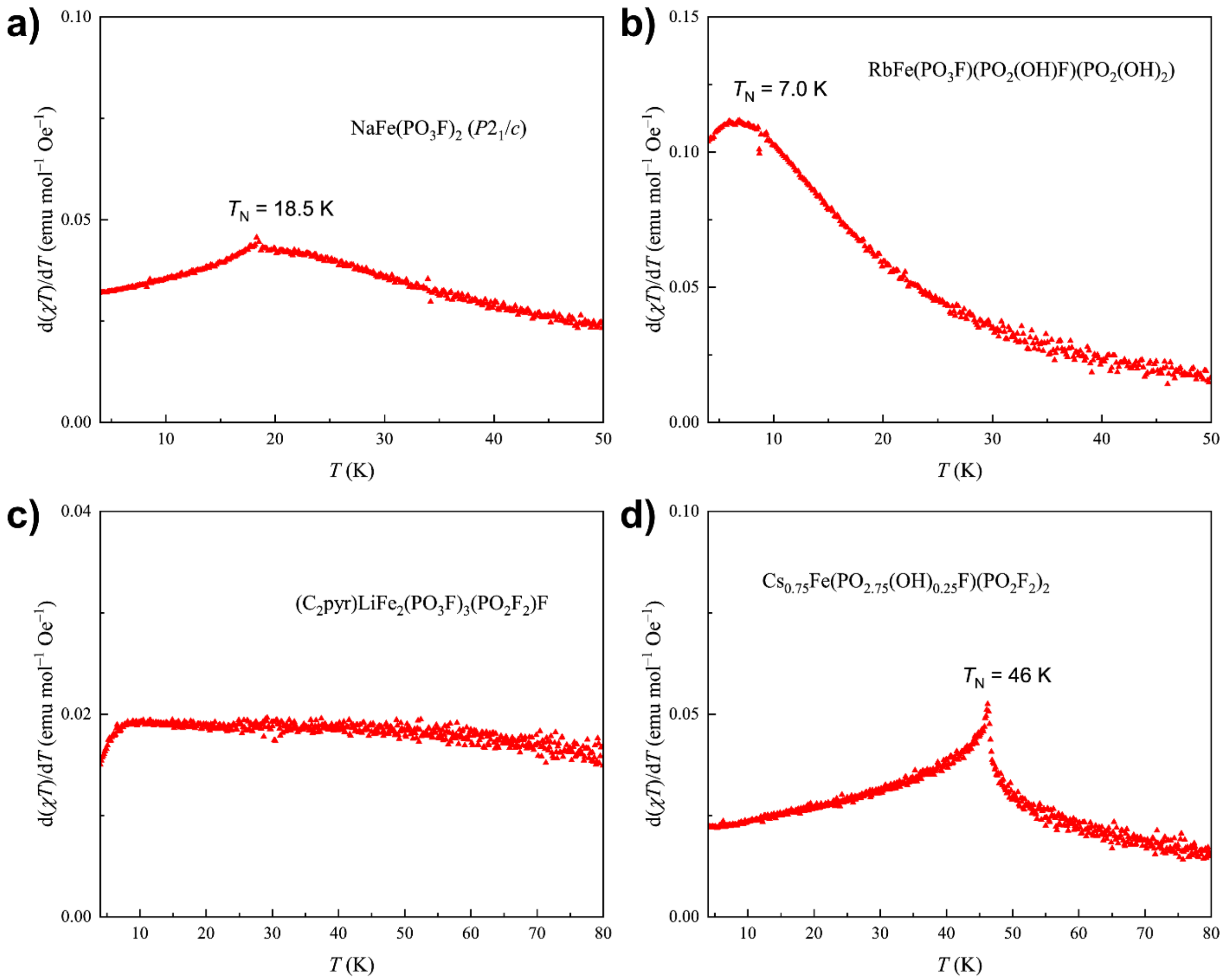

Figure S6. Fisher's heat capacity (from ZFC data) for **1b** (a), **2** (b), **3** (c), and **5** (d).

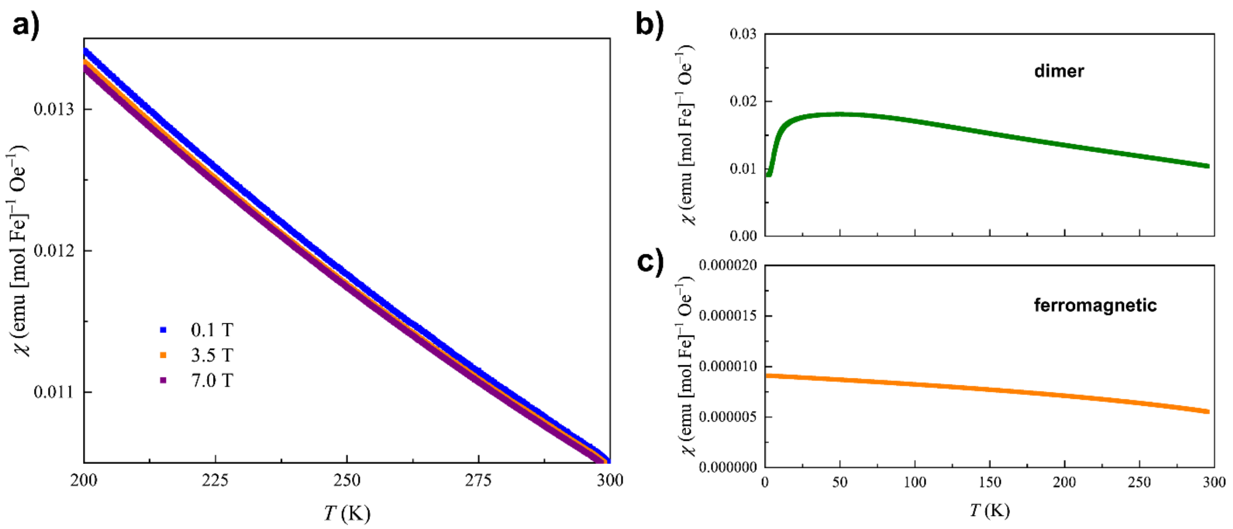

Figure S7. Temperature dependence of magnetic susceptibility (T = 200–300 K) for $(C_2pyr)LiFe_2(PO_3F)_3(PO_2F_2)F$ (**3**), measured under different fields in the field-cooled (FC) regime (a). Dimer (b) and ferromagnetic (c) contributions evaluated with equation (1) in the main text. The given magnetic susceptibility values are calculated with respect to the amount of Fe in $(C_2pyr)LiFe_2(PO_3F)_3(PO_2F_2)F$.

Table S3. ∠Fe–P–Fe angles (°) in orthorhombic (**1a**) and monoclinic (**1b**) $NaFe(PO_3F)_2$.

**1a**	∠Fe1–P1–Fe2	∠Fe2–P1–Fe2	∠Fe1–P2–Fe1	∠Fe1–P2–Fe2	∠Fe2–P3–Fe2
	93.992(44)	153.757(59)	155.221(52)	93.733(44)	101.153(48)
	96.958(46)			96.967(44)	101.564(49)
					153.483(58)
**1b**	∠Fe–P1–Fe	∠Fe–P2–Fe			
	100.936(26)	93.348(27)			
	101.152(26)	95.312(27)			
	154.759(32)	153.704(34)			

Table S4. Crystallographic data and details of structure refinements.

Compound	1a□	2	3	4	5
**CCDC**	2141973	2141974	2141975	2141976	2141977
**Formula**	$NaFeP_2O_6F_2$	$RbFeP_3H_3O_{10}F_2$	$LiFe_2P_4C_7H_{10}NO_{11}F_6$	$LiFeP_2O_5F_4$	$Cs_{0.75}H_{0.25}FeP_3O_7F_5$
**Formula weight, g·mol^{-1}**	274.78	435.25	640.68	280.73	455.69
**T (K)**	297(2)	298(2)	299(2)	295(2)	298(2)
**Crystal system**	Orthorhombic	Monoclinic	Monoclinic	Orthorhombic	Tetragonal
SG	*Fdd*2	$P2_1/c$	$P2_1/c$	$P2_12_12_1$	*I*4/*mcm*
***a*, Å**	9.728(1)	7.645(1)	21.8782(9)	6.4286(2)	18.159(2)
***b*, Å**	42.246(5)	14.801(3)	9.6563(4)	7.6240(3)	18.159(2)
***c*, Å**	8.506(1)	9.518(2)	19.0223(6)	13.8321(6)	12.874(2)
***β*, °**		107.962(6)	100.368(1)		
***V*, Å^3**	3495.4(8)	1024.5(3)	3953.1(3)	677.93(4)	4245.3(9)
Z	24	4	8	4	16
**Density (g/cm^3)**	3.133	2.822	2.153	2.750	2.852
**μ (mm^{-1})**	3.244	6.735	1.901	2.762	4.500
***F*(000)**	3192	836	2528	540	3416
**Index ranges**	$-13 \le h \le 9$	$-10 \le h \le 10$	$-28 \le h \le 28$	$-9 \le h \le 7$	$-25 \le h \le 23$
	$-57 \le k \le 60$	$-21 \le k \le 21$	$-12 \le k \le 12$	$-10 \le k \le 11$	$-25 \le k \le 25$
	$-12 \le l \le 12$	$-13 \le l \le 13$	$-24 \le l \le 23$	$-13 \le l \le 21$	$-18 \le l \le 18$
**Measured reflections**	6623	19324	83875	7840	23316
**Unique reflections**	2604	3143	8875	2567	1671
**Observed reflections**	2329	2474	8292	2378	1566
**Number of parameters**	170	165	574	116	132
**R_{int}**	0.0355	0.0609	0.0474	0.0324	0.0374
***R*, *wR* (all, observed)**	0.0362, 0.0862	0.0363, 0.0681	0.0860, 0.2035	0.0316, 0.0738	0.0445, 0.1189
**$\Delta\rho_{max}$ = (e·Å^{-3})**	1.096	0.737	1.151	0.667	0.934
**$\Delta\rho_{min}$ = (e·Å^{-3})**	–1.062	–0.836	–0.944	-0.585	-0.781
